# Diagnostic Yield of Comprehensive Etiologic Evaluation in Young Adults with Ischemic Stroke: Toward a Phenotype-Driven Approach

**DOI:** 10.3390/jcm15166205

**Published:** 2026-08-11

**Authors:** Aishah Ibrahim Albakr, Alia Alokley, Feras AlSulaiman, Mustafa Ahmed Alqarni, Saud A. Alnaaim, Ali Hafiz Alhashim, Mohammed Alshurem, Abrar J. Alwaheed, Safi G. Alqatari, Erum Sharif, Azra Zafar, Kawther Mohammed Hadhiah, Foziah Jabbar Alshamrani, Rizwana Shahid, Ammar S. Bukhamsin, Fahad Hammad F. Alrayes, Rahaf Marhoom Alsaadi, Farah Abdullah Alsaqr

**Affiliations:** 1 Department of Neurology, College of Medicine, Imam Abdulrahman Bin Faisal University, Dammam 34212, Saudi Arabia; fasulaiman@iau.edu.sa (F.A.); mqarni@iau.edu.sa (M.A.A.); ahalhashem@iau.edu.sa (A.H.A.); mshurem@iau.edu.sa (M.A.); emshariff@iau.edu.sa (E.S.); azafar@iau.edu.sa (A.Z.); khadhiah@iau.edu.sa (K.M.H.); fshamrani@iau.edu.sa (F.J.A.); rshahid@iau.edu.sa (R.S.); asbukhamsin@iau.edu.sa (A.S.B.); fhalrayes@iau.edu.sa (F.H.F.A.); ralsaadi_@outlook.com (R.M.A.); farah622b@gmail.com (F.A.A.); 2 Clinical Neurosciences Department, College of Medicine, King Faisal University, Al-Ahsa 31982, Saudi Arabia; aalokley@kfu.edu.sa (A.A.); salnaim@kfu.edu.sa (S.A.A.); 3 Department of Internal Medicine, College of Medicine, Imam Abdulrahman Bin Faisal University, Dammam 34212, Saudi Arabia; ajwaheed@iau.edu.sa (A.J.A.); sagqatari@iau.edu.sa (S.G.A.)

**Keywords:** young adult, ischemic stroke, embolic stroke of undetermined source, patent foramen ovale, antiphospholipid syndrome, autoimmune disease, phenotype-driven evaluation, Saudi Arabia

## Abstract

**Background/Objectives**: Young-onset ischemic stroke shows substantial etiologic heterogeneity. However, the value and diagnostic yield of advanced cardiac and autoimmune evaluations in young adults remain uncertain. **Methods**: Patients aged 25–55 years admitted with acute ischemic stroke to King Fahd University Hospital in Saudi Arabia between January 2020 and December 2025 were included. Evaluation followed a multidisciplinary young-stroke pathway, incorporating neurovascular imaging, cardiac assessment, and targeted autoimmune and rheumatologic investigations. A Clinically Meaningful Diagnostic Yield (CMDY) was defined as a diagnostic finding that resulted in etiologic reclassification, management modification, or changes to secondary prevention strategy. **Results**: A total of 480 patients were included (median age, 40 years; 67.7% men). CMDY was observed in 200 (41.7%) patients. Among 79 patients initially classified as having embolic stroke of an undetermined source, 35 (44.3%) were assigned a determined etiologic mechanism following evaluation and follow-up. Patients with undetermined stroke etiology decreased from 16.5% after first-pass evaluation to 9.2% after diagnostic completion. Patent foramen ovale/atrial septal defect-associated stroke mechanisms were identified in 61 patients (12.7%), autoimmune/inflammatory stroke mechanisms in 26 patients (5.4%), and atrial fibrillation-related cardioembolic stroke in 18 patients (3.8%). Clinically relevant mechanisms were identified in 169 patients (35.2%) whose primary etiologic classification remained unchanged. Independent predictors of CMDY were hypertension, prior stroke or transient ischemic attack, multi-territory infarction, and cortical infarction. **Conclusions**: Etiologic evaluation yielded clinically meaningful findings in over two-fifths of young adults with ischemic stroke and influenced etiologic assessment, treatment, and secondary prevention. Diagnostic yield was greatest among patients with prior cerebrovascular events and cortical or multi-territory infarctions, supporting a selective, phenotype-driven approach that warrants prospective multicenter validation.

## 1. Introduction

Young-onset ischemic stroke is increasingly recognized as a distinct clinical entity characterized by a prolonged disease trajectory and substantial long-term health consequences [1,2]. Unlike older patients with stroke, affected individuals frequently survive for decades after the index event, exposing them to extended periods of recurrent vascular risk, chronic secondary prevention therapy, and cumulative disabilities. Long-term studies have demonstrated recurrence rates approaching 20% at 10 years, whereas mortality remains significantly higher than that observed in age- and sex-matched populations [3,4]. In addition to physical disability, young stroke survivors commonly experience persistent cognitive, psychological, social, and occupational sequelae, including fatigue, anxiety, depression, fear of recurrence, sexual dysfunction, diminished self-efficacy, loss of employment, and impaired social participation [5,6]. Given the substantial personal and societal burden associated with stroke during the most productive years of life, accurate determination of stroke etiology is of particular clinical importance, as it directly influences prognosis, recurrence prevention, and long-term management strategies.

Although conventional vascular risk factors, including hypertension, diabetes mellitus, dyslipidemia, smoking, and obesity, remain important contributors and are becoming increasingly prevalent among younger adults, the etiologic profile of young-onset ischemic stroke differs substantially from that observed in older populations [1,4]. In addition to premature atherosclerotic disease, young adults exhibit a broader spectrum of causative mechanisms, including cervical artery dissection, Patent Foramen Ovale (PFO), occult cardioembolism, inherited and acquired thrombophilia, Antiphospholipid Syndrome (APS), systemic autoimmune disorders, Central Nervous System (CNS) vasculitis, pregnancy-related complications, and exposure to hormonal therapies [1,7,8]. Regional evidence from Saudi Arabia similarly demonstrates marked etiologic heterogeneity among patients younger than 50 years, underscoring the complexity of diagnostic evaluation in this population [9]. Despite substantial advances in neurovascular imaging, cardiac monitoring, thrombophilia testing, and biomarker-based investigations, a considerable proportion of young adults continue to be classified as having a cryptogenic or Embolic Stroke of Undetermined Source (ESUS) following comprehensive diagnostic assessment [3]. This persistent diagnostic uncertainty highlights the need for improved etiologic characterization and optimized classification strategies for young patients with stroke.

Cardiac abnormalities are among the most clinically actionable mechanisms of young-onset ischemic stroke [10]. PFO has attracted particular attention because of its high prevalence among younger patients with cryptogenic stroke. However, distinguishing an incidental finding from a causal mechanism remains challenging. Contemporary causal attribution frameworks integrate clinical characteristics, neuroimaging findings, and high-risk anatomical features using tools such as the Risk of Paradoxical Embolism (RoPE) score, Morphologic Stroke Factors of PFO (MorPFO) score, and PFO-Associated Stroke Causal Likelihood (PASCAL) classification. Recent pooled analyses of randomized trials have demonstrated substantial heterogeneity in the benefits of transcatheter PFO closure, with the most significant observed in patients with high-risk PFO features and a high probability of PFO-related stroke [11,12]. Moreover, conventional vascular risk factors remain prevalent even among patients with high-risk PFO anatomy, suggesting that stroke causation is frequently multifactorial rather than exclusively attributable to a paradoxical embolism [13]. Comprehensive cardiac evaluations, including prolonged rhythm monitoring and transthoracic and transesophageal echocardiography, further improve the detection of occult cardioembolic sources that may be missed during routine investigations [14,15,16].

Autoimmune and inflammatory disorders constitute a second group of potentially treatable stroke mechanisms. APS, Systemic Lupus Erythematosus (SLE), Sjögren’s syndrome, and other autoimmune rheumatic diseases may promote cerebral ischemia via thrombosis, vasculopathy, accelerated atherosclerosis, and immune-mediated endothelial injury [8,17,18,19]. Population-based data indicate that autoimmune inflammatory rheumatic diseases are associated with an increased risk of ischemic stroke at a younger age, particularly in patients with SLE and systemic vasculitis [17,20]. Establishing such a diagnosis may alter antithrombotic therapy, justify immunomodulatory treatment, and influence long-term surveillance strategies. Nevertheless, the role of broad autoimmune screening remains controversial because positive serological findings, particularly low-titer Antinuclear Antibody (ANA) positivity, are common in the general population and may not represent clinically actionable disease. To improve diagnostic specificity, contemporary classification frameworks for APS, SLE, and primary Sjögren’s syndrome provide standardized approaches for disease identification in both clinical and research settings [18,19,21].

Genetic contributors are increasingly recognized in young patients, particularly those with high rates of consanguinity and inherited disorders [22]. A recent Saudi exome sequencing study identified population-specific variants associated with susceptibility to ischemic stroke, highlighting the potential contribution of rare and novel genetic determinants to cerebrovascular risk [23]. Beyond genomics, emerging biomarker-based approaches may further refine etiologic classification by facilitating discrimination among competing stroke mechanisms [24]. Together, these developments support a precision medicine approach for young-onset ischemic stroke that integrates clinical, imaging, cardiac, autoimmune, and molecular data to improve etiologic attribution and guide individualized management.

This study aimed to determine the clinically meaningful diagnostic yield of a comprehensive etiologic evaluation in young adults with ischemic stroke, with particular emphasis on cardiac and autoimmune/rheumatologic investigations. We further evaluated its impact on etiologic classification, secondary prevention strategies, and subsequent management decisions, and identified clinical and neuroimaging characteristics associated with a higher likelihood of clinically actionable findings.

## 2. Materials and Methods

### 2.1. Study Design, Setting, and Ethical Approval

We conducted a retrospective cohort study using data from a prospectively maintained stroke registry at King Fahd University Hospital, a tertiary comprehensive stroke center affiliated with Imam Abdulrahman Bin Faisal University, Dammam, Saudi Arabia. Their institutional pathway for young-onset ischemic stroke evaluation aligns with the contemporary American Heart Association/American Stroke Association (AHA/ASA) guidelines for secondary stroke prevention and Canadian Stroke Best Practice Recommendations for stroke management and secondary prevention [10,25,26]. Demographic, clinical, laboratory, imaging, cardiac, and outcome data were prospectively collected as part of routine clinical care.

The study was approved by the Institutional Review Board (IRB-2026-01-0406), and informed consent was waived due to the retrospective use of de-identified data. This study was conducted in accordance with the Declaration of Helsinki and reported in accordance with the Strengthening the Reporting of Observational Studies in Epidemiology (STROBE) guidelines.

### 2.2. Study Population

Although the initial registry screening included patients aged 18–55 years, eligibility for the present study was restricted a priori to adults aged 25–55 years. Patients aged 18–24 years were therefore excluded from the analytical cohort because they did not meet the predefined age criterion. The study sample was determined by the number of consecutive adult eligible patients aged 25–55 years who were admitted for acute ischemic stroke during the predefined study period from January 2020 to December 2025. An upper age limit of 55 years was selected because most contemporary studies of young-onset ischemic stroke define young adulthood as ≤55 years, facilitating comparison with international cohorts and etiologic studies [1,4,6,7]. The inclusion criteria included acute ischemic stroke (confirmed via a stroke specialist assessment plus neuroimaging evidence of infarction) and sufficient diagnostic information to permit etiologic classification according to the study framework. Patients with transient neurological symptoms without infarction, stroke mimics, alternative non-vascular diagnoses, primary intracerebral hemorrhage, cerebral venous sinus thrombosis, or insufficient data were excluded before cohort assembly.

As this was a retrospective registry-based cohort, no target sample size was specified a priori, and all patients meeting the predefined inclusion and exclusion criteria were included. This consecutive inclusion approach was intended to maximize representativeness and minimize selection bias.

**Figure 1:**
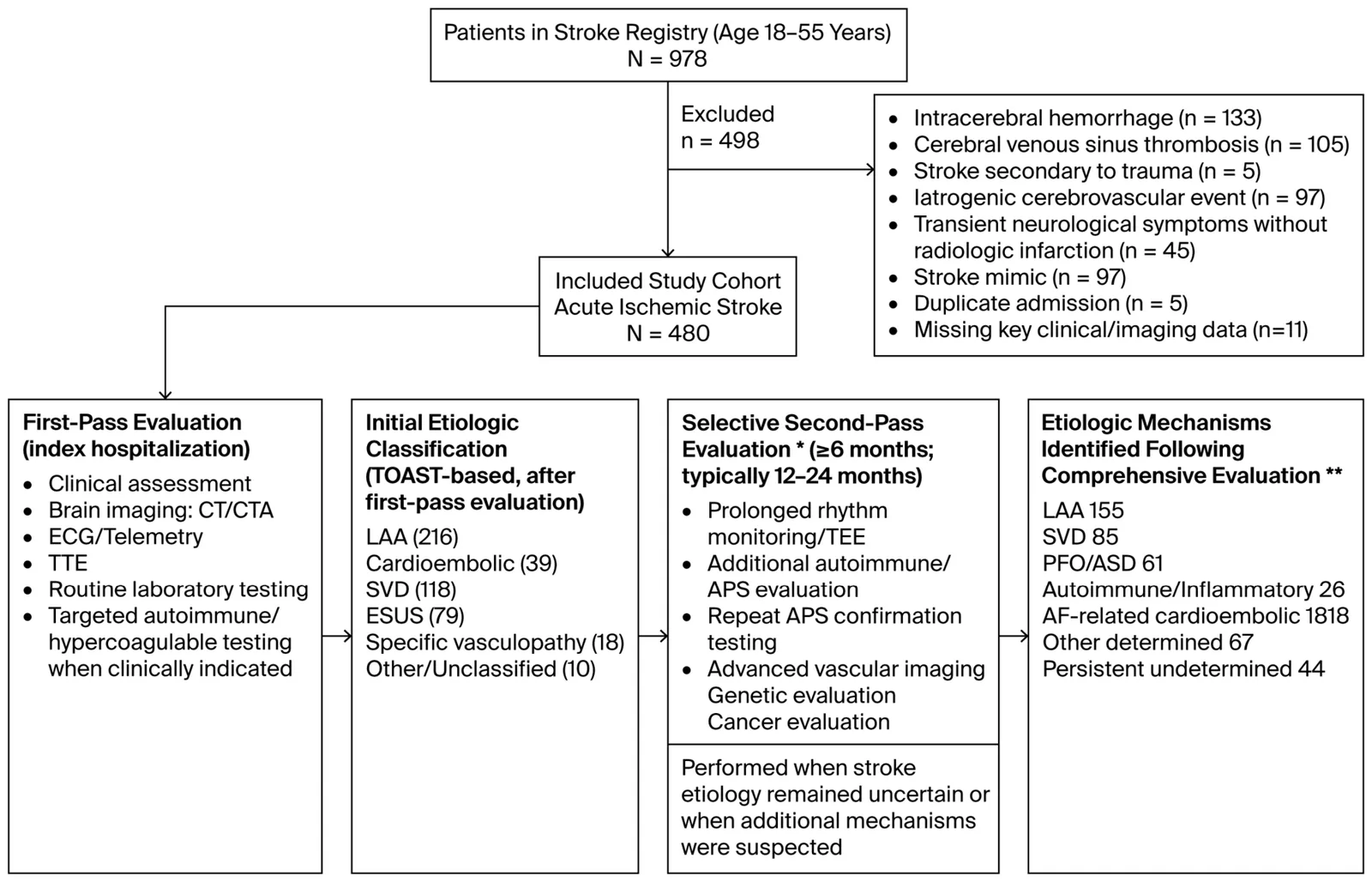
The study cohort, staged diagnostic evaluation, and etiologic classification workflow. Categories are not mutually exclusive. More than one clinically relevant stroke mechanism may have been identified for patients during comprehensive evaluation; therefore, the category totals may not equal the overall study population. The diagram distinguishes between first-pass investigations performed during index hospitalization and second-pass investigations performed selectively based on clinical indications. ESUS represents a provisional working diagnosis following first-pass evaluation and remains subject to revision during longitudinal follow-up. * Selective second-pass evaluation was performed ≥6 months after the index hospitalization (typically at 12–24 months) when the stroke etiology remained uncertain or additional mechanisms were suspected. ** Final etiologic classification was assigned following comprehensive evaluation. Abbreviations: AF, atrial fibrillation; APS, antiphospholipid syndrome; ASD, atrial septal defect; ESUS, embolic stroke of undetermined source; LAA, large-artery atherosclerosis; PFO, patent foramen ovale; SVD, small-vessel disease; TEE, transesophageal echocardiography; TOAST, Trial of Org 10172 in Acute Stroke Treatment.

The cohort comprised all consecutive eligible patients identified during the study period. No patients were selected based on their clinical outcomes or follow-up status. Patients with previously established autoimmune, rheumatologic, hypercoagulable, malignant, or other stroke-relevant conditions were not excluded. They were classified according to their final stroke mechanism after completion of the diagnostic evaluation. For the CMDY analysis, pre-existing diagnoses were not considered diagnostic findings. A pre-existing condition was counted as CMDY only if additional investigations provided new information relevant to the stroke index, demonstrated active disease contributing to the event, resulted in etiologic reclassification, or led to a change in clinical management. The study workflow is shown in Figure 1.

### 2.3. Data Collection and Stroke Characterization

The demographic variables included age and sex. The clinical variables included Body Mass Index (BMI), obesity (BMI ≥ 30 kg/m^2^), hypertension, diabetes mellitus, dyslipidemia, smoking, and prior stroke or Transient Ischemic Attack (TIA). Additional young-onset stroke risk factors included migraine with aura, pregnancy or postpartum status, hormonal therapy exposure [1,7], active malignancy, recent infection, substance use, and family history of young-onset stroke or autoimmune diseases.

Stroke severity at presentation was assessed using the National Institutes of Health Stroke Scale (NIHSS) and analyzed as a continuous variable [27].

Functional status was assessed using the Modified Rankin Scale (mRS) at baseline and 3-month follow-up [26]. Follow-up data were obtained from stroke and rehabilitation outpatient clinic visits and supplemented by electronic medical record review, in which mRS scores were routinely documented as part of institutional clinical practice. Etiologic reclassification was based on all available investigations and longitudinal follow-up data recorded in the institutional stroke registry and was assigned according to the Trial of Org 10172 in Acute Stroke Treatment (TOAST) classification.

### 2.4. Etiologic Evaluation Protocol

All patients underwent a standardized evaluation in accordance with the contemporary AHA/ASA [10] and Canadian [25] guidelines. Initial assessments included non-contrast brain Computed Tomography (CT), intracranial and extracranial vascular imaging, 12-lead Electrocardiography (ECG), inpatient telemetry, routine laboratory testing, vascular risk factor assessment, and transthoracic echocardiography. Brain Magnetic Resonance imaging (MRI) was performed during the index admission or early follow-up in most patients and contributed to classification in these cases.

Infarct location, cortical involvement, multi-territory infarction, and stroke subtype were determined using all available neuroimaging data. High-resolution vessel-wall MRI and catheter angiography were not routinely performed and were reserved for selected patients with suspected vasculitis, arterial dissection, or other non-atherosclerotic vasculopathies. Genetic testing was not performed routinely and was reserved for highly selected patients with clinical, radiological, or familial features suggestive of hereditary stroke syndrome [15,16,28].

Baseline laboratory evaluations included routine hematological, biochemical, coagulation, and vascular risk factor assessments. Advanced cardiac investigations, including transesophageal echocardiography, microbubble contrast studies, and prolonged cardiac rhythm monitoring, were selectively performed when clinically indicated to investigate potential cardioembolic mechanisms [14,15,16]. Autoimmune, thrombophilia, hypercoagulability, and rheumatologic investigations were performed selectively based on stroke characteristics, vascular risk factor burden, recurrent thrombotic events, systemic clinical features, and the presence or absence of an alternative etiologic mechanism [17,27,29].

Data completeness exceeded 95% for the variables included in the multivariate analyses. Autoimmune, thrombophilia, advanced vascular imaging, and genetic investigations were performed selectively rather than routinely; therefore, untested patients cannot be excluded from the corresponding conditions. Consequently, the observed diagnostic yields for these specialized evaluations are likely conservative estimates, and the prevalence of individual etiologic mechanisms cannot be inferred from this cohort.

### 2.5. Staged Diagnostic Evaluation

The diagnostic evaluation was conceptualized as a staged process:

First-pass evaluation consisted of investigations performed during index hospitalization, including clinical assessment, brain and vascular imaging, ECG, inpatient cardiac monitoring, transthoracic echocardiography, and routine laboratory testing. Additional autoimmune and hypercoagulability investigations were performed during the initial evaluation when clinically indicated, particularly in patients with cryptogenic stroke, recurrent thrombotic events, a personal or family history suggestive of thrombophilia, systemic features of autoimmune disease, or an absence of conventional vascular risk factors.

Second-pass evaluation further investigations were performed during follow-up when the stroke etiology remained uncertain, recurrent events occurred, or specific clinical features suggested an alternative mechanism. These tests were performed selectively and included prolonged cardiac rhythm monitoring, transesophageal echocardiography, autoimmune and APS testing, advanced vascular imaging, and selective genetic evaluations.

Etiologic reclassification was defined as a change in the primary stroke etiology identified after the first-pass evaluation compared with the final etiologic assignment after all investigations and longitudinal follow-ups were performed. Etiologic reclassification was analyzed as a secondary outcome. Reclassification could occur between any major etiologic category, including Large-Artery Atherosclerosis (LAA), cardioembolism, Small-Vessel Disease (SVD), ESUS, or other determined etiologies.

### 2.6. Operational Definitions and Etiologic Classification

Stroke subtypes were initially classified according to the (TOAST) criteria [30]. The final etiologic attribution was assigned by the treating stroke neurologist after completion of all available investigations and follow-ups. To support phenotyping, cases were evaluated using the ASCO (A: atherosclerosis; S: small vessel disease; C: cardiac pathology; O: other causes) classification, Causative Classification of Stroke (CCS) system, and the updated ASCOD (A: atherosclerosis; S: small vessel disease; C: cardiac pathology; O: other causes; D: dissection) framework [28,31]. Concordance between the etiologic assignments generated by TOAST, CCS, and ASCOD was moderate to substantial (Cohen’s κ = 0.62).

LAA was defined as ischemic stroke associated with ≥50% stenosis or occlusion of the relevant extracranial or intracranial artery in the absence of a more likely competing mechanism [10,28]. Cardioembolic stroke requires the presence of a major cardiac source, including atrial fibrillation, atrial flutter, intracardiac thrombus, mechanical prosthetic valves, infective endocarditis, or another established high-risk cardioembolic condition. The left ventricular thrombus was considered a definite source of cardioembolic conditions. Reduced ejection fraction or cardiomyopathy in the absence of an intracardiac thrombus was interpreted within the overall clinical context and was not considered sufficient to establish a cardioembolic etiology [10,28,30].

SVD was defined as a lacunar infarct with a compatible clinical syndrome and neuroimaging pattern without evidence of competing large-artery or cardioembolic mechanisms [30,32].

ESUS was defined according to the Cryptogenic Stroke/ESUS International Working Group criteria as a non-lacunar ischemic stroke without ≥50% stenosis in the supplying artery, without a major-risk cardioembolic source, and without another specific cause identified after standard evaluation [33,34,35]. Because ESUS represents a working diagnosis rather than a definitive mechanism, cases were subject to etiologic revision during follow-up [34,35].

Other determined etiologies included uncommon but identifiable stroke mechanisms such as autoimmune or inflammatory vasculopathies, APS and other acquired or inherited hypercoagulable states, arterial dissection, non-atherosclerotic arteriopathies, cancer-associated stroke, genetic or metabolic disorders, and drug-related stroke. These mechanisms were considered the most likely causes after integration of all available clinical, imaging, laboratory, and follow-up data [1,30,31,32,36].

Atrial fibrillation and atrial flutter were documented using a 12-lead ECG, inpatient telemetry, or ambulatory rhythm monitoring. All patients underwent an ECG, 24–48 h of cardiac telemetry, and transthoracic echocardiography during the initial evaluation. Extended ambulatory rhythm monitoring was selectively performed during the follow-up of patients with persistent diagnostic uncertainty or suspected occult cardioembolism [21,25,28,30,37].

Cardiac structural abnormalities, including PFO and ASD, were initially identified using transthoracic echocardiography and further characterized using transesophageal echocardiography when clinically indicated [38,39]. The likelihood of a causal relationship between interatrial communication and stroke index was assessed retrospectively using the RoPE score and PASCAL classification, integrating clinical characteristics, neuroimaging findings, exclusion of competing stroke mechanisms, and the presence of high-risk anatomical features [12,40,41]. PFO- or ASD-associated stroke was assigned as the final primary etiology only when paradoxical embolism was considered the most plausible explanation after reviewing all available clinical, imaging, laboratory, and follow-up data. Defects lacking high-risk anatomical features or those identified in the presence of a more convincing alternative mechanism were considered incidental findings [12,40,41].

APS, SLE, Sjögren syndrome, and rheumatoid arthritis were diagnosed according to the contemporary American College of Rheumatology/European League Against Rheumatism classification criteria [18,19,21,42]. Primary CNS angiitis was diagnosed based on compatible clinical, laboratory, neuroimaging, and angiographic findings, with histopathological confirmation when available and after exclusion of secondary vasculitic and non-vasculitic mimics [43,44]. For APS, positive antiphospholipid antibody results were confirmed by repeat testing for at least 12 weeks after the initial assessment in accordance with international consensus recommendations [19,37].

Investigations that were not performed were coded as “not ordered” and analyzed separately from the negative test results to preserve information regarding diagnostic completeness and patterns of clinical evaluation.

Cortical infarction was defined as an infarction involving the cerebral cortex on Computed Tomography (CT) or MRI. Multi-territory infarction was defined as acute ischemic lesions involving more than one vascular territory on neuroimaging. The cumulative burden of vascular risk was used to characterize the overall vascular risk profile of the cohort [28,30,45].

A cumulative vascular risk score was calculated by assigning one point for each conventional vascular risk factor, including obesity (BMI ≥ 30 kg/m^2^) [46], hypertension [47], diabetes mellitus, dyslipidemia, current smoking, and prior stroke or TIA. Diabetes mellitus was defined according to the American Diabetes Association criteria [48], including a documented diagnosis, use of glucose-lowering medication for diabetes mellitus, a glycated hemoglobin (HbA1c) level ≥6.5%, a fasting plasma glucose level ≥126 mg/dL (7.0 mmol/L), or a 2-h plasma glucose level ≥200 mg/dL (11.1 mmol/L). Obesity was defined as a BMI ≥30 kg/^2^ [46]. Dyslipidemia was defined as low-density lipoprotein cholesterol ≥100 mg/dL (2.6 mmol/L) [49]. The total score ranged from 0 to 6 and was categorized as a low (0–1 points), moderate (2–3 points), or high (≥4 points) vascular risk burden.

### 2.7. Outcome Definitions

#### 2.7.1. CMDY

CMDY was the primary outcome and was defined as the identification of diagnostic information that resulted in a meaningful change in stroke etiologic understanding, clinical management, or long-term patient monitoring. A finding was considered to represent CMDY when it fulfilled at least one of the following criteria: (i) established or substantially modified etiologic classification; (ii) initiation, discontinuation, or modification of secondary prevention therapy; (iii) a procedural intervention, including referral for PFO or ASD closure; or (iv) disease-specific specialist management, surveillance, or long-term follow-up.

CMDY classification was performed retrospectively by applying prespecified operational criteria to clinical decisions, specialist recommendations, treatment modifications, procedural referrals, and follow-up plans documented prospectively during routine patient care. The investigators conducting the retrospective review were not blinded to the general study objectives; however, the underlying etiologic classifications and management decisions had been made by the treating multidisciplinary stroke team before the present study analyses and were not influenced by the study hypothesis. No independent outcome adjudication committee was convened.

The CMDY was assessed across the entire diagnostic pathway. Etiologic reclassification represented only one component of the CMDY; findings of altered management, surveillance, or specialist follow-up without changing the assigned primary stroke mechanism were also considered clinically meaningful [50,51].

Examples of CMDY included newly diagnosed atrial fibrillation, major cardioembolic sources such as left ventricular thrombus, clinically significant PFO or ASD, cervical artery dissection, APS, SLE, and primary CNS angiitis. Findings that did not alter the etiologic attribution, management, or follow-up strategies were not considered clinically meaningful. These included isolated laboratory abnormalities, nonspecific autoantibody positivity, low-titer ANA positivity, isolated inflammatory marker elevation without a confirmed diagnosis, and low-risk PFOs without evidence supporting a causal relationship with the stroke index.

#### 2.7.2. Secondary Outcomes

Secondary outcomes included etiologic reclassification, identification of additional clinically relevant mechanisms that did not alter the primary etiologic classification, and diagnostic efficiency of major evaluation pathways expressed as the Number Needed to Test (NNTx).

Etiologic reclassification was defined as a change in the primary etiologic attribute between the initial classification following the first-pass evaluation and the final classification assigned after the completion of second-pass investigations and longitudinal follow-up. Additional clinically relevant mechanisms were defined as findings with implications for management, secondary prevention, or follow-up that did not replace the assigned primary stroke etiology.

### 2.8. Statistical Analysis

All analyses were performed using R statistical software version 4.5.0 (R Foundation for Statistical Computing, Vienna, Austria). Continuous variables were assessed for normality using the Shapiro–Wilk test. Normally distributed variables are presented as the mean ± standard deviation, whereas non-normally distributed variables are presented as the median and Interquartile Range (IQR). Group comparisons were performed using the independent-samples *t*-test or Mann–Whitney *U* test, as appropriate. Categorical variables are presented as frequencies and percentages and were compared using the chi-square or Fisher’s exact test, as appropriate. All statistical tests were two-sided, and statistical significance was defined as *p* < 0.05.

Baseline demographic, vascular, clinical, imaging, cardiac, autoimmune/rheumatologic, etiologic, and outcome characteristics were compared between patients with and without CMDY. Variables that directly contributed to the definition of CMDY, including clinically significant PFO/ASD, newly detected atrial fibrillation, APS, confirmed autoimmune diagnoses, other management-changing findings, and established stroke mechanisms identified during the diagnostic evaluation, were excluded from the multivariable predictor analyses to minimize incorporation bias and avoid circular inference.

The candidate predictor variables were selected based on their clinical relevance and biological plausibility. To preserve model stability and minimize overfitting, covariate selection was restricted according to events-per-variable considerations, maintaining an approximate ratio of 15:1 [52]. The final model included age, sex, obesity, hypertension, prior stroke or TIA, baseline NIHSS score, cortical infarction, multi-territory infarction, and large-vessel occlusion. The results are reported as adjusted Odds Ratios (aORs) with 95% Confidence Intervals (CIs).

Model discrimination was assessed using the area under the receiver operating characteristic curve (AUC/C-statistic), and model calibration was evaluated using the Hosmer–Lemeshow goodness-of-fit test. Etiologic agreement between classification systems was quantified using Cohen’s κ statistic and interpreted as poor (<0.20), fair (0.20–0.40), moderate (0.41–0.60), substantial (0.61–0.80), or almost perfect (>0.80) [53].

For major diagnostic pathways, the NNTx was calculated as the number of patients undergoing a given diagnostic evaluation divided by the number of CMDY events directly attributable to that pathway.

As registry completeness exceeded 95% for all variables included in the multivariable model, a complete case analysis was performed without imputation. Advanced diagnostic investigations were performed according to clinical indications rather than uniformly across the cohort; therefore, absence of testing was not interpreted as an absence of disease, and the diagnostic yield of specialized investigations may have been underestimated.

## 3. Results

### 3.1. Study Population and Baseline Characteristics

Of the 978 patients aged 18–55 years identified during the initial registry screening, patients aged 18–24 years and those meeting the prespecified clinical exclusion criteria were excluded before assembly of the final analytical cohort. Therefore, 498 were excluded: 133 (13.6%) with primary intracerebral hemorrhage, 105 (10.7%) with cerebral venous sinus thrombosis, 97 (9.9%) with stroke mimics, and 45 (4.6%) with neuroimaging-negative transient symptoms. The remaining exclusion criteria included traumatic or iatrogenic cerebrovascular events, duplicate records, and insufficient data. After applying the eligibility criteria, 480 patients with confirmed acute ischemic stroke were included in the study.

Twenty-eight patients (5.8%) had a previously established autoimmune, rheumatologic, or hypercoagulable-associated condition at presentation and were retained. The median age was 40 years (IQR, 32–48 years), and 325 patients (67.7%) were men. Hypertension was present in 128 patients (26.7%), diabetes in 79 patients (16.5%), dyslipidemia in 104 patients (21.7%), current smoking in 115 patients (24.0%), and obesity in 164 patients (34.2%). CMDY was identified in 200 (41.7%) patients, who were more likely to have hypertension (37.5% vs. 18.9%, *p* < 0.001) and prior stroke/TIA (11.0% vs. 4.6%, *p* = 0.014). No significant differences were observed in age, sex, BMI, obesity, diabetes, dyslipidemia, or smoking status (Table 1).

### 3.2. Stroke Characteristics and Neuroimaging Findings

The median baseline NIHSS score was four (IQR, 2–8) and did not differ between groups (*p* = 0.960). The pre-stroke mRS score was one (IQR, 1–2) in both groups (*p* = 0.385); this reflects mild pre-existing disability in a subset of patients, including those with prior stroke and other chronic neurological or medical conditions. The following neuroimaging findings were analyzed independently: Cortical infarction was more frequent in patients with CMDY than in those without CMDY (44.0% vs. 32.9%, *p* = 0.017). Multi-territory infarction was also more common in the CMDY group (12.0% vs. 5.7%, *p* = 0.022). Lacunar infarction, posterior circulation involvement, and large-vessel occlusion did not differ significantly between the groups (Table 2).

### 3.3. Diagnostic Yield of Cardiac Evaluation

First-pass cardiac evaluation identified major cardioembolic sources in 39 patients, who were subsequently classified as having cardioembolic stroke. A Left ventricular thrombus was considered a definitive cardioembolic source. By contrast, a reduced ejection fraction in the absence of intracardiac thrombus was not considered sufficient alone, when interpreted within the overall clinical context, to establish a cardioembolic etiology.

Second-pass cardiac evaluations, including transesophageal echocardiography and prolonged rhythm monitoring, identified additional clinically relevant mechanisms. Cardiac rhythm monitoring detected atrial fibrillation in 18 patients and other clinically significant arrhythmias in 15 patients, resulting in 33 clinically actionable rhythm monitoring findings (6.9%). PFO- or ASD-associated stroke was assigned as the final primary etiology in 61 patients (12.7%), of whom 60 were referred for closure evaluation or intervention (Table 3).

### 3.4. Diagnostic Yield of Autoimmune, Rheumatologic, and Hypercoagulable Evaluation

Autoimmune or hypercoagulation testing was performed in 367 patients (76.5%). ANA positivity was common (151 patients, 31.5%) but did not differ between the CMDY and non-CMDY groups (32.5% vs. 30.7%, *p* = 0.752). Isolated ANA positivity is frequently observed in the absence of clinically significant autoimmune disease.

In contrast, disease-specific autoimmune and antiphospholipid antibodies were significantly more frequent in patients with CMDY. Elevated inflammatory markers (erythrocyte sedimentation rate/C-reactive protein), anti-dsDNA antibodies, extractable nuclear antigen antibodies, anticardiolipin antibodies, and anti-β2-glycoprotein I antibodies were all associated with a higher likelihood of CMDY.

Initial antiphospholipid antibody positivity was identified in 64 patients (13.3%). Following repeated testing and clinical correlation analyses, persistent APS was confirmed in 17 patients (3.5%). Autoimmune, rheumatologic, and hypercoagulable evaluations yielded 37 clinically meaningful findings (7.7%), resulting in management modifications, specialist follow-up, or etiologic clarification. An autoimmune or inflammatory mechanism was assigned as the primary stroke etiology in 26 patients (5.4%), including APS, SLE, primary CNS angiitis, Sjögren’s syndrome, rheumatoid arthritis, and other autoimmune disorders (Table 4).

### 3.5. Clinical Impact of Comprehensive Diagnostic Evaluation

CMDY was observed in 200 (41.7%) patients. Among those initially classified as ESUS, etiologic reclassification was performed in 35 patients (44.3% in the ESUS subgroup and 7.3% in the overall cohort). This finding indicates that more than half of the clinically meaningful findings influenced management, monitoring, or specialist follow-up without changing the assigned primary stroke mechanism.

PFO/ASD-associated findings resulted in a referral for closure evaluation or intervention in 60 (12.5%) patients. By contrast, autoimmune, rheumatologic, and hypercoagulable findings led to clinically meaningful changes in the management of 37 (7.7%) patients. Eight patients with recurrent ischemic stroke were subsequently found to have clinically relevant mechanisms that were not identified during the initial evaluation, resulting in the modification of their secondary prevention strategies. The clinical impact categories were not mutually exclusive.

The diagnostic yield was greatest among patients initially classified as ESUS. Of the 79 patients in this category, 35 (44.3%) were assigned a determined etiologic mechanism following comprehensive evaluation, whereas 44 (55.7%) were classified as having a persistent undetermined etiology (Table 5).

The primary etiologic attribution remained unchanged in 155 of 216 patients (71.8%) with LAA, 104 of 118 patients (88.1%) with SVD, and 63 of 67 patients (94.0%) with other determined etiologies. Additional clinically relevant mechanisms that did not replace the primary etiologic classification were identified in 61 patients (28.2%) with LAA, 104 patients (88.1%) with SVD, and four patients (6.0%) with other determined etiologies. PFO/ASD-associated stroke occurred in 61 patients (12.7%), autoimmune/inflammatory stroke in 26 patients (5.4%), and atrial fibrillation-related cardioembolic stroke in 18 patients (3.8%).

**Figure 2:**
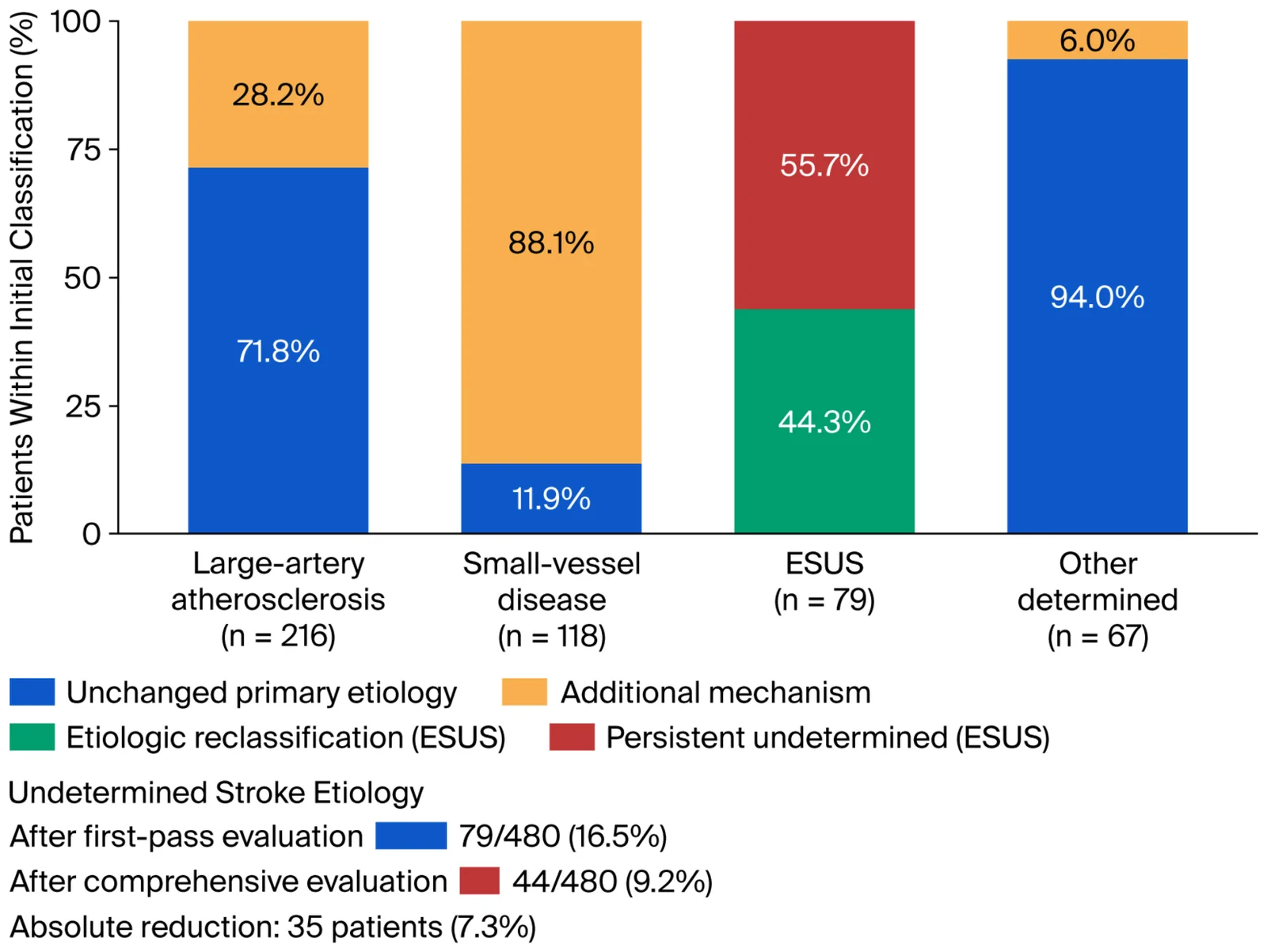
Diagnostic outcomes following comprehensive evaluation and longitudinal follow-up according to initial stroke classification. Bars show the proportion of patients who had unchanged primary etiologic classification and an additional clinically relevant stroke mechanism. Etiologic reclassification was performed for patients initially classified as having ESUS or persistent undetermined etiology. Additional clinically relevant mechanisms were identified in patients whose primary etiologic classification remained unchanged; therefore, these categories are not mutually exclusive. Abbreviations: ESUS, embolic stroke of undetermined source.

The number of patients with undetermined stroke etiology decreased from 79 (16.5%) after the first-pass evaluation to 44 (9.2%) after completion of the longitudinal diagnostic evaluation (Figure 2). The major etiologic mechanisms identified during the comprehensive evaluation are summarized in Table 6.

Supplementary Figure S1 illustrates the transition from the initial TOAST-based classification to the final etiologic attribution following a comprehensive evaluation, including a reduction in persistent undetermined stroke etiology from 16.5% to 9.2%.

### 3.6. Diagnostic Efficiency of Major Diagnostic Pathways

Diagnostic efficiency varied across the evaluation pathways (Table 7). The number needed to test (NNTx) was 4.4 for the PFO/ASD evaluation pathway, 14.1 for autoimmune/rheumatologic evaluation, and 26.7 for cardiac rhythm evaluation. These values represent the number of patients who underwent a given diagnostic pathway per final etiologic diagnosis directly attributable to that evaluation.

### 3.7. Predictors of CMDY

**Figure 3:**
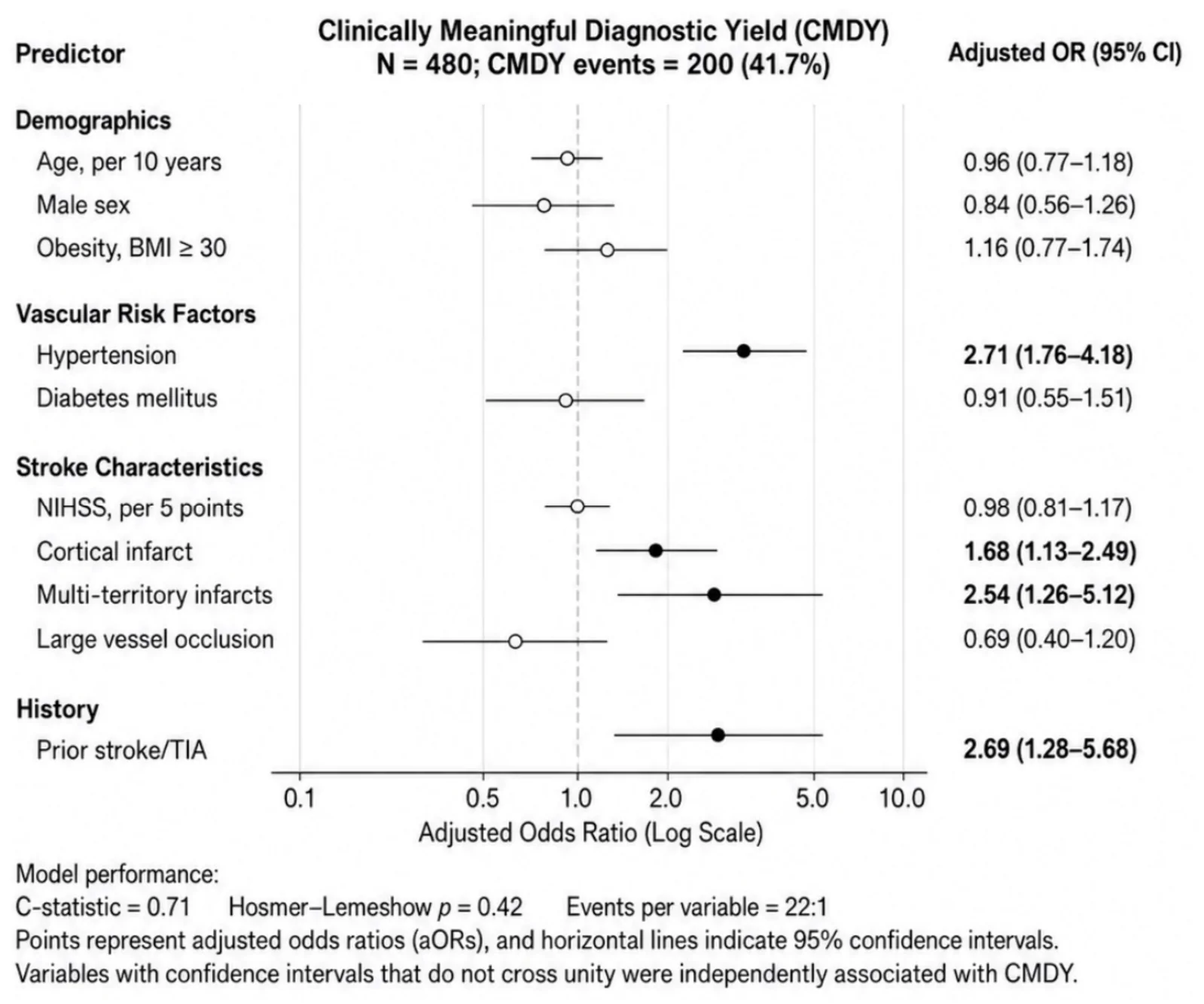
Independent predictors of clinically meaningful diagnostic yield identified using multivariable logistic regression. Forest plot showing adjusted odds ratios (aORs) and 95% confidence intervals for candidate predictors of CMDY. Filled circles indicate statistically significant predictors, whereas open circles indicate nonsignificant predictors. The vertical dashed line represents an aOR of 1.0 (no association). Model discrimination was moderate (C-statistic = 0.71), and calibration was acceptable (Hosmer–Lemeshow *p* = 0.42). Abbreviations: aOR, adjusted odds ratio; BMI, body mass index; CI, confidence interval; CMDY, clinically meaningful diagnostic yield; NIHSS, National Institutes of Health Stroke Scale; TIA, transient ischemic attack.

Multivariate logistic regression identified hypertension, prior stroke or TIA, multi-territory infarction, and cortical infarction as independent predictors of CMDY (Table 8; Figure 3). Hypertension demonstrated the strongest association, followed by prior stroke/TIA, multi-territory infarction, and cortical infarction.

No independent associations were observed with age, sex, obesity, diabetes mellitus, baseline NIHSS score, or large-vessel occlusion. The multivariate model demonstrated acceptable discrimination (C-statistic, 0.71) and good calibration (Hosmer–Lemeshow test, *p* = 0.42).

## 4. Discussion

### 4.1. Principal Findings and Clinical Impact

In this single-center cohort of young adults with ischemic stroke, comprehensive evaluation produced CMDY in 41.7% of patients, whereas etiologic reclassification represented only one component of this clinical impact. Our findings support the accumulating evidence that ESUS and cryptogenic stroke should be viewed as working diagnoses rather than definitive etiologic entities [34,35,54]. Among the 79 patients initially classified as having ESUS, 35 (44.3%) were assigned a determined mechanism after second-pass investigations and longitudinal reassessment, reducing persistent undetermined etiology from 16.5% to 9.2%. These findings support viewing ESUS as a provisional working diagnosis in selected young adults and demonstrate that diagnostic value also includes treatment modification, procedural referral, specialist management, and identification of additional mechanisms that may coexist with the primary etiologic classification.

The coexistence of vascular, cardiac, inflammatory, and prothrombotic abnormalities also highlights the limitations of strictly single-cause classification systems in young adults. A broader phenotypic framework may better represent the biological complexity of young-onset stroke while preserving assignment of the most likely primary mechanism.

Similar findings were reported in the NOR-FIB study, in which a probable underlying cause, most commonly atrial fibrillation and atrial flutter, was identified in 43% of patients with initial cryptogenic stroke or TIA after 12 months of follow-up [50]. The authors concluded that cryptogenic stroke commonly reflects an incomplete stage of etiologic characterization rather than a definitive diagnosis and should be viewed as a working diagnosis pending further investigation and follow-up. Similarly, Aarnio et al. demonstrated that the incidence of cryptogenic stroke decreased over time in young adults, whereas definite cardioembolic mechanisms became increasingly recognized during long-term follow-up [51].

The heterogeneous nature of ESUS may help explain these findings. Data from the NAVIGATE-ESUS trial showed that many patients had more than one potential embolic source, including atrial cardiomyopathy, structural cardiac abnormalities, atherosclerotic disease, and other competing mechanisms [54]. In this setting, ESUS may be viewed as an incomplete etiologic evaluation, rather than a distinct disease entity [50,51]. Consistent with this concept, our study showed that systematic second-pass investigations and follow-up frequently identified previously unrecognized stroke mechanisms, including structural cardiac abnormalities, APS, autoimmune diseases, inflammatory vasculopathy, and other uncommon causes, with important implications for secondary prevention and long-term management [45,50,51]. Overall, these findings highlight the value of longitudinal etiologic reassessment in young adults with initially unexplained stroke and support a dynamic approach to stroke classification that accommodates evolving diagnostic information over time.

Figure 2 shows that the comprehensive evaluation’s diagnostic impact varied with the initial stroke classification. Although the principal benefit among patients initially classified as ESUS was etiologic reclassification, the predominant benefit among patients with established etiologic subtypes was the identification of additional clinically relevant mechanisms. Additional mechanisms were identified in 28.2% of patients initially classified as having LAA and 88.1% of those initially classified as having SVD.

These findings highlight the limitations of traditional single-cause classification systems in young adults with ischemic stroke. Although the traditional TOAST classification system is based on assigning a single dominant etiologic category, many patients exhibit coexisting vascular, cardiac, inflammatory, or prothrombotic features that contribute to the overall stroke phenotype and may have important implications for risk stratification, secondary prevention, and long-term management [30]. In contrast, the ASCO classification, later refined into the ASCOD framework, was developed to identify the multifactorial nature of stroke by characterizing multiple coexisting disease processes within the same patient [31,55,56].

Taken together, these observations support a broader phenotypic approach to stroke assessment in young adults, in which coexisting mechanisms are recognized alongside primary etiologic attributes, rather than viewed as competing alternatives. Such an approach may better reflect the biological complexity of stroke in younger populations and facilitate individualized management strategies.

### 4.2. Predictors and Phenotype-Informed Diagnostic Evaluation of CMDY

In the multivariate analysis, hypertension, prior stroke or TIA, cortical infarction, and multi-territory infarction were independently associated with CMDY. These associations are biologically plausible. Hypertension and recurrent cerebrovascular events may act as indicators for patients with a greater burden of vascular disease or previously unrecognized competing stroke mechanisms. By contrast, infarct patterns involving the cortex or multiple vascular territories may reflect underlying embolic, paradoxical, inflammatory, or prothrombotic processes that are not readily apparent during the initial evaluation [34,35,45,51,57]. In contrast, demographic characteristics, including age and sex, were not independently associated with diagnostic yield after adjustment for clinical and imaging features.

These findings suggest that the CMDY varies among young adults with ischemic stroke and is more strongly associated with clinical and neuroimaging phenotypes rather than demographic characteristics alone [50,51,57]. Accordingly, a phenotype-informed approach may help prioritize advanced etiologic investigations and optimize resource utilization while ensuring comprehensive evaluation of patients most likely to benefit continues. However, given the retrospective design, these observations should be considered hypothesis-generating and require prospective validation.

**Figure 4:**
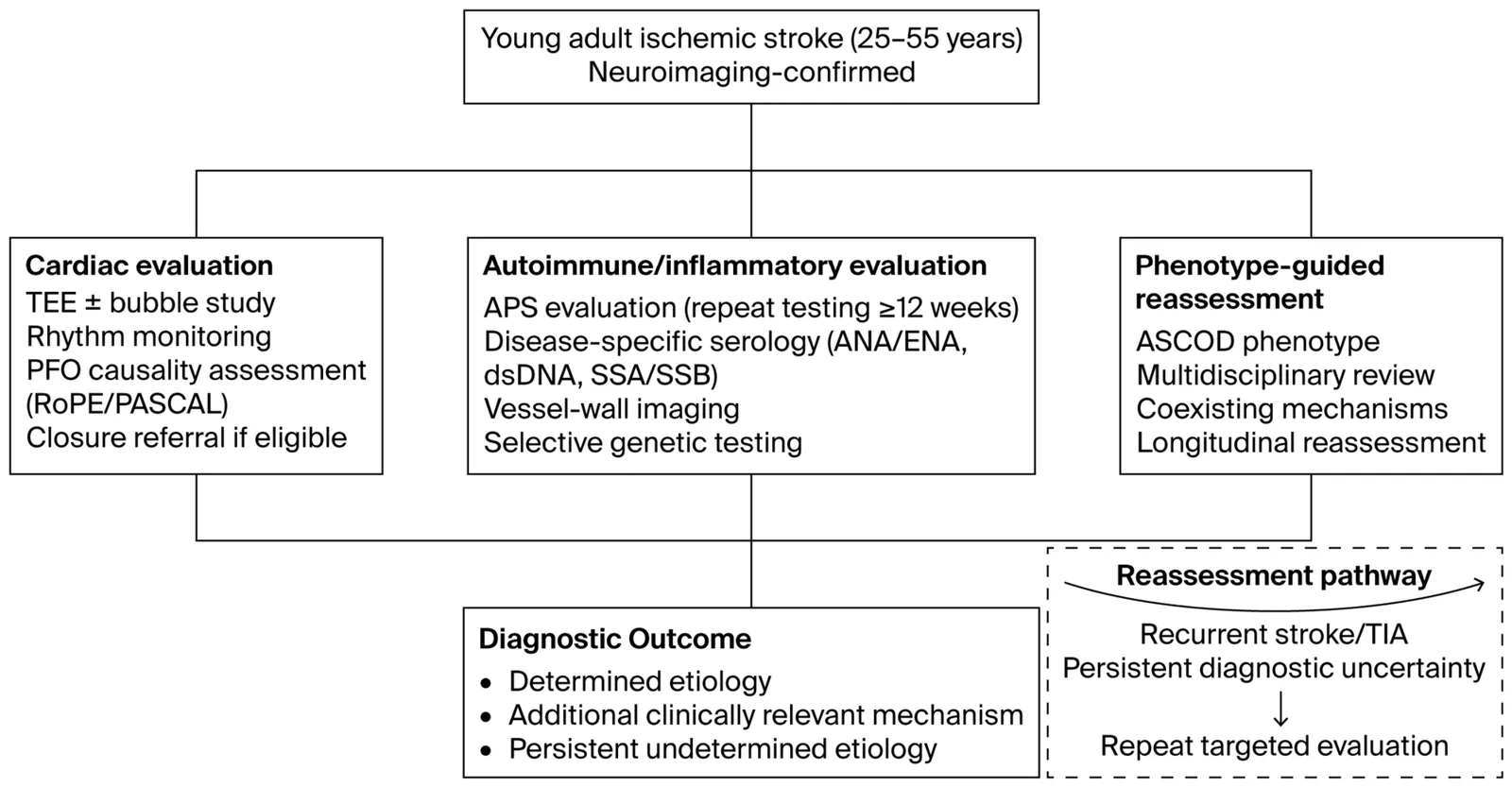
Proposed phenotype-driven diagnostic evaluation framework for young-onset ischemic stroke. Following confirmation of acute ischemic stroke, patients undergo targeted evaluation through three complementary pathways: (1) cardiac investigation, (2) autoimmune and inflammatory evaluation, and (3) phenotypic reassessment. Patients experiencing recurrent ischemic events or persistent undetermined stroke etiology may undergo repeat targeted evaluation and longitudinal reassessment. The framework is derived from the findings of the present study and contemporary guideline recommendations. It is intended to support clinical reasoning and future prospective validation rather than serve as a prescriptive diagnostic algorithm. Abbreviations: APS, antiphospholipid syndrome; ASCOD, atherosclerosis, small-vessel disease, cardiac pathology, other causes, and dissection phenotyping system; dsDNA, anti-double-stranded DNA antibody; ENA, extractable nuclear antigen; PFO, patent foramen ovale; RoPE, risk of paradoxical embolism; SSA/SSB, Sjögren syndrome-related antigens A and B; TEE, transesophageal echocardiography.

Building on these observations, Figure 4 proposes a phenotype-informed framework for the etiologic evaluation of young adults with ischemic stroke. This framework integrates the findings of the present study with the existing literature and emphasizes a phenotype-guided approach in the cardiac, autoimmune/inflammatory, and neuroimaging domains. Recognizing that stroke mechanisms frequently overlap and that etiologic attribution may evolve during follow-up, this framework is intended to support clinical reasoning rather than dictate management. Therefore, it should be viewed as a conceptual model that may inform future research and warrants prospective validation before routine clinical implementation.

### 4.3. Cardiac Diagnostic Pathway

Cardiac evaluation provided etiologic clarification, where PFO/ASD represents the most common cardiac mechanism, accounting for 12.7% of the final primary etiologic classifications. The high proportion of PFO/ASD-related strokes observed in our cohort emphasizes the clinical relevance of evaluating interatrial communication within a broader clinical and neuroimaging context, particularly when the overall stroke phenotype is suggestive of paradoxical embolism [12,38,41,58,59,60]. The diagnostic contribution of transesophageal echocardiography was substantial and consistent with previous studies, demonstrating its incremental value in identifying occult cardioembolic sources and structural cardiac abnormalities that are not apparent on routine transthoracic imaging, frequently resulting in clinically meaningful changes in management [16,61]. Correspondingly, PFO/ASD evaluation demonstrated the highest diagnostic efficiency in our cohort, with an NNTx value of 4.4.

Prolonged rhythm monitoring identified atrial fibrillation in 3.8% of patients. This relatively modest detection rate is consistent with evidence demonstrating that occult atrial fibrillation becomes increasingly prevalent with advancing age and is less frequently identified in younger adults with cryptogenic stroke [62,63]. Notably, atrial fibrillation was detected in a small proportion of patients despite an inconclusive initial evaluation, supporting the value of prolonged cardiac rhythm monitoring. This observation suggests that stroke etiology may become apparent over time and that selected patients may benefit from extended cardiac surveillance when the initial evaluation remains inconclusive. Although the diagnostic efficiency of rhythm monitoring was lower (NNTx 26.7), identifying occult atrial fibrillation has major therapeutic implications because it fundamentally alters secondary prevention strategies from antiplatelet therapy to long-term anticoagulant therapy.

### 4.4. Autoimmune and Rheumatologic Evaluation

Autoimmune and rheumatologic evaluations were performed in 367 (76.5%) patients: an underlying stroke mechanism was identified in 5.4% of the cohort, and persistent APS was confirmed in 3.5% of patients following repeat testing and clinical correlation. These findings align with the contemporary evidence demonstrating that autoimmune and rheumatic disorders contribute substantially to cerebrovascular diseases in younger adults, particularly SLE, systemic vasculitis, and APS. Recent population-based analyses involving more than 22 million individuals have demonstrated an increased risk of cardiovascular and cerebrovascular diseases across a broad spectrum of autoimmune disorders, with the relative risk being greatest in younger patients and among those with SLE [64]. Together, these observations reinforce the importance of considering autoimmune mechanisms in young adults with otherwise unexplained ischemic stroke and support the use of targeted rheumatologic evaluation when routine investigations are inconclusive [17,65,66].

However, our findings highlight the limitations of indiscriminate serological screening. ANA positivity was common but not associated with CMDY, whereas disease-specific autoantibodies and confirmed autoimmune diagnoses were strongly associated with actionable findings. Broadly, nonspecific ANA screening has limited diagnostic utility because ANA positivity is relatively common in healthy individuals and lacks specificity for systemic autoimmune diseases [67,68]. In contrast, disease-specific autoantibodies—including anti-dsDNA, ENA, anticardiolipin, and anti-β2-glycoprotein I antibodies—were more strongly associated with clinically actionable findings, suggesting that phenotype-guided testing focused on disease-specific autoantibodies may provide greater diagnostic yield than broad serologic screening [67,68,69]. This observation is consistent with contemporary laboratory guidelines, which recommend ANA testing only when there is clinical suspicion of connective tissue disease and advocate targeted follow-on testing with disease-specific autoantibodies based on the clinical phenotype and initial serologic findings [69].

The distinction between isolated antiphospholipid antibody positivity and persistent APS warrants particular emphasis, as misinterpreting a single positive antiphospholipid antibody result may lead to incorrect attribution of stroke etiology and inappropriate long-term anticoagulant therapy. In our cohort, 13.3% of patients had a positive initial antiphospholipid antibody test result; however, only 3.5% fulfilled the criteria for persistent APS after repeat testing and clinical correlation. This finding underscores the importance of adhering to the established APS classification and diagnostic criteria, which require persistent antiphospholipid antibody positivity on at least two occasions, separated by a minimum of 12 weeks [19,70]. Failure to distinguish transient antibody positivity from persistent APS may substantially overestimate the contribution of APS to ischemic stroke and expose patients to unnecessary long-term anticoagulant therapy.

The diagnostic efficiency of autoimmune testing should be interpreted within its clinical context. Although autoimmune/rheumatologic evaluation demonstrated an intermediate NNTx of 14.1, this should not be interpreted as a measure of clinical importance compared to other investigations. Similar to prolonged cardiac rhythm monitoring, autoimmune testing identifies a relatively small subset of patients. However, those identified often have diagnoses with major implications for secondary stroke prevention and long-term management, including APS, SLE, and primary CNS vasculitis. Consequently, differences in NNTx should not be construed as differences in clinical value. Rather, NNTx reflects the prevalence of the conditions investigated and supports the selective application of testing in patients with an appropriate pre-test probability. This approach is consistent with contemporary diagnostic guidelines, including those of the 2023 ACR/EULAR APS Classification Criteria and British Society for Hematology, both of which advocate targeted testing based on clinical features and disease-specific suspicion [19,69].

Such selectivity is particularly important because the identification of diagnoses such as atrial fibrillation, APS, SLE, or primary CNS vasculitis has major implications for secondary stroke prevention and long-term management. Therefore, the diagnostic yield should be interpreted alongside the clinical significance of the diagnoses identified, rather than in isolation [71].

### 4.5. Persistent Undetermined Stroke and Genetics

Despite comprehensive evaluation, 9.2% of patients remained without a definitive etiologic attribution. Similar findings have been reported in contemporary young-onset stroke cohorts. The SECRETO study demonstrated that a substantial proportion of young adults with ischemic stroke do not have a clearly identifiable cause despite extensive phenotyping and comprehensive diagnostic investigation [72]. Likewise, data from the ESUS Global Registry showed that approximately one-sixth of patients with ischemic stroke fulfill the criteria for embolic stroke of undetermined source despite undergoing standard diagnostic evaluation. This highlights the persistent challenge of establishing a definitive etiologic diagnosis in a subset of patients [73,74].

Although the proportion of patients with persistently undetermined stroke in our cohort was lower than that reported in many contemporary studies [75,76], these findings highlight the ongoing challenges of establishing a definitive stroke mechanism in a subset of young adults despite comprehensive diagnostic evaluation. Potential explanations include atrial cardiopathy without documented atrial fibrillation, non-stenotic but clinically relevant atherosclerotic disease, occult inflammatory or prothrombotic disorders, and genetic cerebrovascular conditions that are difficult to identify using conventional diagnostic approaches [35,54].

Genetic testing was not performed systematically in the present study and was generally reserved for selected patients with suggestive clinical, radiological, or familial features. Consequently, an underlying genetic cause may have remained undetected in some patients who were classified as having an undetermined stroke. Emerging Saudi genomic studies have identified novel variants associated with ischemic stroke and suggested that inherited cerebrovascular disorders may account for a proportion of otherwise unexplained strokes in this population [23]. Future prospective registries incorporating structured genetic evaluations may improve etiologic attribution and advance precision medicine approaches for young-onset stroke.

### 4.6. Saudi-Specific Context

Saudi registry data on young-onset ischemic stroke are limited. The most directly comparable prior study was the Babtain cohort, which demonstrated substantial heterogeneity in stroke etiology among patients younger than 50 years, despite including less comprehensive diagnostic evaluation [9]. The present study extends these observations by applying a more granular diagnostic pathway, incorporating longitudinal follow-up, and evaluating both etiologic and clinical management outcomes. Although the two studies share the limitations inherent to single-center retrospective designs, our findings provide additional insights into the diagnostic complexity of young-onset stroke in Saudi Arabia. Furthermore, our results are in concordance with those of international cohorts, including the SECRETO study, ESUS-focused cohorts and registries, Find-AF, and other young-stroke registries [2,3,13,33,73,77], supporting the broader applicability of a phenotype-driven diagnostic approach within Saudi and Gulf populations [75,76].

### 4.7. Strengths and Limitations

This study has several strengths. The study included a large, well-characterized cohort of young adults with ischemic stroke who underwent comprehensive evaluation within a multidisciplinary young-stroke program. This study reflects real-world clinical practice, where investigations are guided by clinical presentations and evolving diagnostic information rather than a rigid research protocol. Another strength is the integration of both etiologic and clinical management outcomes within a single primary endpoint, allowing the impact of diagnostic investigations to be assessed beyond test positivity or changes in stroke classification. The parallel evaluation of the cardiac, autoimmune, rheumatologic, and hypercoagulable pathways also enabled the assessment of their complementary contributions to diagnostic clarification and patient management.

This study has some limitations that warrant consideration. First, it was retrospective and conducted at a single tertiary comprehensive stroke center, which limited generalizability and introduced a potential selection bias. Second, the final etiologic attribution was based on clinical assessments by treating stroke neurologists, and no independent adjudication committee was convened; therefore, residual diagnostic uncertainty remains possible, particularly in patients with multiple potentially relevant stroke mechanisms. Third, CMDY was intentionally designed as a composite measure of the overall clinical impact of comprehensive etiologic evaluation. Although its components were defined using prespecified operational criteria, they represent clinically distinct outcomes and may differ in importance and susceptibility to clinical judgment. The investigators conducting the retrospective review were not blinded to the general study objectives, and no independent adjudication committee was convened; therefore, residual classification bias cannot be excluded. A sensitivity analysis using a more restrictive CMDY definition was not performed. Future prospective studies should evaluate individual CMDY components, apply alternative composite definitions, and incorporate blinded independent adjudication. Fourth, diagnostic investigations were not performed uniformly across the cohorts. Autoimmune, thrombophilia, hypercoagulability, genetic, and advanced vascular imaging investigations were performed according to clinical indications rather than systematically; therefore, their true diagnostic yield may have been underestimated. Fifth, the relatively small number of recurrent stroke events limited our ability to evaluate the association between specific diagnostic findings and long-term recurrence. Six, the high-risk PFO and ESUS subgroups were modest in size; therefore, subgroup-specific estimates should be interpreted with caution. Finally, systematic genetic testing was not routinely performed, which is a particularly relevant limitation in Saudi Arabia, where consanguinity may increase the prevalence of monogenic cerebrovascular disorders. The study was also not sufficiently powered to assess the effects of clinically meaningful individual diagnostic findings on recurrence or functional outcomes.

### 4.8. Clinical Implications

The findings of this study support a selective, phenotype-driven approach for the etiologic evaluation of young adults with ischemic stroke. Patients with hypertension, prior cerebrovascular events, cortical infarction, or multi-territory infarction demonstrated a greater likelihood of CMDY and may therefore derive particular benefit from expanded investigations. Importantly, the clinical value of comprehensive evaluation extends beyond changes in the primary etiologic classification and frequently influences secondary prevention strategies, procedural decisions, specialist referrals, and long-term follow-up.

Figure 4 presents a phenotype-driven diagnostic framework that synthesizes the principal findings of this study using a structured, clinical approach. Rather than relying on a linear exclusion-based workup, the framework emphasizes parallel, targeted evaluation across cardiac, autoimmune/inflammatory, and phenotypic domains while acknowledging that multiple stroke mechanisms may coexist and etiologic attribution may evolve over time. The framework is intended to support clinical reasoning rather than function as a prescriptive algorithm. Prospective multicenter validation is required to determine whether implementing this approach improves the diagnostic yield, risk stratification, treatment decisions, and long-term outcomes of these patients.

## 5. Conclusions

Comprehensive cardiac, autoimmune, rheumatologic, and hypercoagulable evaluations yielded clinically meaningful findings in more than two-fifths of young adults with ischemic stroke, substantially influencing etiologic assessment, secondary prevention, and long-term management. The clinical value of the evaluation extended beyond changes observed in the primary etiologic classification and included the identification of additional clinically relevant mechanisms that informed risk stratification, treatment decisions, and follow-up care. The diagnostic yield was greatest among patients with hypertension, prior cerebrovascular events, and cortical or multi-territory infarction, whereas isolated nonspecific serologic abnormalities demonstrated limited clinical utility. These findings support a selective, phenotype-driven, multidisciplinary approach to etiologic evaluation and underscore the need for classification frameworks that can accommodate the multifactorial nature of stroke pathogenesis in young adults. Prospective multicenter validation is warranted before this framework can be incorporated into formal clinical guidelines.

## Figures and Tables

**Table 1:** Baseline demographic, clinical, and vascular characteristics according to CMDY (*n* = 480).

Variable	Total (*n* = 480)	CMDY (*n* = 200)	No CMDY (*n* = 280)	*p*-Value
**Demographics**				
Age, years, median [IQR]	40.0 [32.0, 48.0]	40.0 [32.0, 47.0]	40.0 [32.0, 48.0]	0.814
Male sex	325 (67.7%)	133 (66.5%)	192 (68.6%)	0.704
BMI, kg/m^2^, median [IQR]	27.8 [23.9, 31.3]	28.4 [24.3, 31.3]	27.1 [23.7, 31.2]	0.298
Obesity (BMI ≥ 30 kg/m^2^)	164 (34.2%)	73 (36.5%)	91 (32.5%)	0.416
**Vascular risk factors**				
Hypertension	128 (26.7%)	75 (37.5%)	53 (18.9%)	<0.001
Diabetes mellitus	79 (16.5%)	36 (18.0%)	43 (15.4%)	0.519
Dyslipidemia	104 (21.7%)	47 (23.5%)	57 (20.4%)	0.477
Current smoking	115 (24.0%)	48 (24.0%)	67 (23.9%)	1.000
Prior stroke/TIA	35 (7.3%)	22 (11.0%)	13 (4.6%)	0.014
**Additional young-onset stroke risk factors**				
Migraine with aura	33 (6.9%)	15 (7.5%)	18 (6.4%)	0.784
OCP/hormonal therapy *	58 (12.1%)	17 (8.5%)	41 (14.6%)	0.058
Active cancer	37 (7.7%)	12 (6.0%)	25 (8.9%)	0.311
Recent infection	11 (2.3%)	3 (1.5%)	8 (2.9%)	0.374
Substance use (cocaine/amphetamine)	58 (12.1%)	21 (10.5%)	37 (13.2%)	0.449
Family history of young stroke	51 (10.6%)	20 (10.0%)	31 (11.1%)	0.822
Family history of autoimmune disease	15 (3.1%)	8 (4.0%)	7 (2.5%)	0.506
**Baseline stroke characteristics**				
Baseline NIHSS, median [IQR]	4.0 [2.0, 8.0]	4.0 [2.0, 8.0]	4.0 [2.0, 8.0]	0.960
Pre-stroke mRS, median [IQR]	1.0 [1.0, 2.0]	1.0 [1.0, 2.0]	1.0 [1.0, 2.0]	0.385

* Female-specific variable. Values are median [IQR] or *n* (%). *p*-values: Mann–Whitney *U* (continuous); χ^2^ or Fisher’s exact (categorical). Abbreviations: BMI, body mass index; CMDY, clinically meaningful diagnostic yield; IQR, interquartile range; mRS, modified Rankin Scale; NIHSS, National Institutes of Health Stroke Scale; OCP, oral contraceptive pill; TIA, transient ischemic attack.

**Table 2:** Stroke characteristics, neuroimaging findings, and functional outcomes according to CMDY (*n* = 480).

Variable	Total (*n* = 480)	CMDY (*n* = 200)	No CMDY (*n* = 280)	*p*-Value
Cortical infarct	180 (37.5%)	88 (44.0%)	92 (32.9%)	0.017
Lacunar/small subcortical infarct	104 (21.7%)	48 (24.0%)	56 (20.0%)	0.349
Multi-territory infarcts	40 (8.3%)	24 (12.0%)	16 (5.7%)	0.022
Posterior circulation stroke	85 (17.7%)	34 (17.0%)	51 (18.2%)	0.824
Large-vessel occlusion	72 (15.0%)	29 (14.5%)	43 (15.4%)	0.897

Values are presented as numbers (%). *p*-values compare patients with and without CMDY using χ^2^ or Fisher’s exact tests, as appropriate. Cortical infarcts are ischemic lesions involving the cerebral cortex. Lacunar/small subcortical infarcts were defined according to the standard neuroimaging criteria for small-vessel disease. Multi-territory infarcts were defined as acute ischemic lesions involving more than one vascular territory. Large-vessel occlusion was defined as intracranial occlusion of a major cerebral artery on vascular imaging. All were analyzed independently. Abbreviations: CMDY, clinically meaningful diagnostic yield.

**Table 3:** Clinically relevant findings from cardiac evaluation (*n* = 480).

Variable	*n* (%)
PFO/ASD-associated stroke mechanism	61 (12.7)
Atrial fibrillation on cardiac rhythm monitoring	18 (3.8)
Other clinically significant arrhythmias	15 (3.1)
Any clinically actionable rhythm monitoring finding	33 (6.9)

PFO/ASD-associated stroke was assigned only when paradoxical embolism was considered the most likely stroke mechanism following multidisciplinary clinical evaluation and review of neuroimaging and cardiac findings. Rhythm monitoring findings were considered clinically actionable when they resulted in a change in diagnosis, secondary prevention strategy, clinical management, or follow-up. Abbreviations: ASD, atrial septal defect; PFO, patent foramen ovale.

**Table 4:** Autoimmune, rheumatologic, and hypercoagulable evaluation according to CMDY.

Variable	Total (*n* = 480)	CMDY (*n* = 200)	No CMDY (*n* = 280)	*p*-Value
**Autoimmune testing**				
Autoimmune workup performed	367 (76.5%)	159 (79.5%)	208 (74.3%)	0.223
ANA performed	367 (76.5%)	159 (79.5%)	208 (74.3%)	0.223
ANA titer				0.914
ANA-negative, among those tested	216/367 (58.9%)	94/159 (59.1%)	122/208 (58.7%)	
ANA-positive, among those tested	151/367 (41.1%)	65/159 (49.9%)	86/208 (41.3%)	
Low-titer ANA, among positive results	125/151 (82.8%)	54/65 (83.1%)	71/86 (82.6%)	
High-titer ANA, among positive results	26/151 (17.2%)	11/65 (16.9%)	15/86 (17.4%)	0.752
Isolated ANA positivity without clinically significant autoimmune disease	110 (22.9%)	39 (19.5%)	71 (25.4%)	0.163
**Inflammatory markers and autoimmune serologies**				
Elevated ESR and/or CRP	69 (14.4%)	42 (21.0%)	27 (9.6%)	<0.001
dsDNA-positive	14 (2.9%)	12 (6.0%)	2 (0.7%)	0.002
ENA-positive	19 (4.0%)	16 (8.0%)	3 (1.1%)	<0.001
Lupus anticoagulant-positive	43 (9.0%)	22 (11.0%)	21 (7.5%)	0.444
Anticardiolipin antibody-positive	19 (4.0%)	15 (7.5%)	4 (1.4%)	0.002
β2-glycoprotein I antibody-positive	20 (4.2%)	15 (7.5%)	5 (1.8%)	0.006
Initial aPL positivity	64 (13.3%)	34 (17.0%)	30 (10.7%)	0.041

Values are presented as numbers (%). *p*-values compare patients with and without CMDY using χ2 or Fisher’s exact tests, as appropriate. ANA results are reported among patients in whom ANA testing was performed. ANA positivity was classified according to the highest titer recorded during the diagnostic evaluation. Low- and high-titer categories are reported among patients with a positive ANA result. Patients without ANA testing were retained as a separate “not ordered” category in the underlying dataset and were not classified as ANA-negative. ANA positivity was categorized as the highest titer recorded during evaluation. Isolated ANA positivity refers to positive ANA serology and no evidence of a clinically relevant autoimmune disorder after specialist assessment. Elevated inflammatory marker levels were defined as elevated ESR and/or CRP levels. Initial aPL positivity reflects the results of the first test and does not confirm APS. APS diagnosis required persistent antibody positivity on repeat testing performed at least 12 weeks apart, together with compatible clinical criteria according to the established international classification criteria. Positive aPLs included lupus anticoagulant, anticardiolipin, and anti-β2-glycoprotein I antibodies. Abbreviations: ANA, antinuclear antibody; aPL, antiphospholipid antibody; APS, antiphospholipid syndrome; CMDY, clinically meaningful diagnostic yield; CRP, C-reactive protein; dsDNA, anti-double-stranded DNA antibody; ENA, extractable nuclear antigen; ESR, erythrocyte sedimentation rate.

**Table 5:** Clinical impact of comprehensive diagnostic evaluation (*n* = 480).

Outcome	*n* (%)
CMDY	200 (41.7)
Additional diagnostic finding beyond initial classification	204 (42.5)
ESUS reclassified	35 (44.3)
Persistent ESUS	44 (55.7)

Categories are not mutually exclusive; individual patients may contribute to more than one outcome category. The percentages of ESUS reclassification and persistent ESUS were calculated among patients initially classified as having ESUS (*n* = 79) as the denominator rather than the overall study cohort (*n* = 480). Abbreviations: CMDY, clinically meaningful diagnostic yield; ESUS, embolic stroke of undetermined source.

**Table 6:** Primary and additional etiologic mechanisms identified following comprehensive diagnostic evaluation (*n* = 480).

Identified Etiologic Mechanism	*n* (%)
Large-artery atherosclerosis	155 (32.3)
Small-vessel disease	85 (17.7)
PFO/ASD-associated stroke mechanism	61 (12.7)
Autoimmune/inflammatory stroke mechanism	26 (5.4)
Atrial fibrillation-related cardioembolic stroke	18 (3.8)
Cervical artery dissection	35 (7.3)
Other determined stroke mechanism	67 (13.9)
Persistent undetermined etiology	44 (9.2)

Percentages represent the proportion of the entire cohort (*n* = 480) in which each etiologic mechanism was identified. Categories are not mutually exclusive because patients may have had more than one clinically relevant etiologic mechanism identified during the evaluation; therefore, percentages do not sum up to 100%. The number of patients with undetermined stroke etiology decreased from 79 (16.5%) after the initial evaluation to 44 (9.2%) after the completion of comprehensive diagnostic evaluation and longitudinal follow-up. Abbreviations: ASD, atrial septal defect; PFO, patent foramen ovale.

**Table 7:** Number needed to identify a clinically relevant etiologic diagnosis.

Pathway	Patients Tested, *n*	Etiologic Diagnoses Identified, *n*	NNTx
PFO/ASD evaluation	270	61	4.4
Autoimmune/rheumatologic evaluation	367	26	14.1
Cardiac rhythm evaluation	480	18	26.7

NNTx was calculated as the number of patients undergoing a given diagnostic evaluation divided by the number of final etiologic diagnoses directly attributable to that pathway. For autoimmune/rheumatologic evaluation, only cases in which an autoimmune or inflammatory disorder was assigned as the primary stroke mechanism were counted, and isolated serologic abnormalities without etiologic attribution were excluded. For cardiac rhythm evaluation, only patients who were ultimately classified as having atrial fibrillation-related cardioembolic stroke were included. For PFO/ASD evaluation, only patients in whom paradoxical embolism was determined to be the most likely stroke mechanism following multidisciplinary clinical assessment were included. Lower NNTx values indicate greater diagnostic efficiency. Abbreviations: ASD, atrial septal defect; NNTx, number needed to test; PFO, patent foramen ovale.

**Table 8:** Independent predictors of CMDY following comprehensive diagnostic evaluation.

Variable	aOR	95% CI	*p*-Value
**Hypertension**	**2.71**	**1.76–4.18**	**<0.001**
**Prior stroke/TIA**	**2.69**	**1.28–5.68**	**0.009**
**Multi-territory infarct**	**2.54**	**1.26–5.12**	**0.009**
**Cortical infarct**	**1.68**	**1.13–2.49**	**0.011**
Obesity	1.16	0.77–1.74	0.415
Large-vessel occlusion	0.69	0.40–1.20	0.707
Age (per 10-year increase)	0.96	0.77–1.18	0.611
Baseline NIHSS (per 5-point increase)	0.98	0.81–1.17	0.650
Male sex	0.84	0.56–1.26	0.782
Diabetes mellitus	0.91	0.55–1.51	0.721

Multivariable logistic regression was performed to identify factors independently associated with clinically meaningful diagnostic yield (CMDY). Variables were selected a priori based on their clinical relevance and univariate associations. An aOR > 1 indicates an increased likelihood of CMDY. Statistically significant associations are shown in bold. Abbreviations: aOR, adjusted odds ratio; CI, confidence interval; CMDY, clinically meaningful diagnostic yield; NIHSS, National Institutes of Health Stroke Scale; TIA, transient ischemic attack.

## Data Availability

The datasets generated and/or analyzed during the current study are not publicly available because they contain confidential patient information. Public sharing of individual-level data is restricted under the conditions of Institutional Review Board approval (IRB-2026-01-0406) and the research ethics, privacy, and confidentiality policies of Imam Abdulrahman Bin Faisal University and King Fahad University Hospital.

## Supplementary Materials

The following supporting information can be downloaded at https://www.mdpi.com/article/10.3390/jcm15166205/s1. Figure S1: First-pass versus final etiologic attribution following comprehensive diagnostic evaluation; Table S1: Impact of comprehensive diagnostic evaluation according to initial stroke classification.
