# Supplementary material for: Diagnostic Yield of Comprehensive Etiologic Evaluation in Young Adults with Ischemic Stroke: Toward a Phenotype-Driven Approach
Source: J Clin Med. 2026 Aug 11;15(16):6205. doi: 10.3390/jcm15166205 (PMC13513393; doi:10.3390/jcm15166205)
Supplement: Supplementary file 1 [file jcm-15-06205-s001.zip › jcm-4451558-supplementary.pdf]

Supplementary Materials

Supplementary Table S1. Impact of Comprehensive Diagnostic Evaluation According to Initial Stroke Classification.

Initial classification	Outcome	n (%)
Large-artery atherosclerosis	Unchanged primary etiology	155 (71.8)
Large-artery atherosclerosis	Additional clinically relevant mechanism	61 (28.2)
Small-vessel disease	Unchanged primary etiology	14 (11.9)
Small-vessel disease	Additional clinically relevant mechanism	104 (88.1)
ESUS	Reclassified to determined etiology	35 (44.3)
ESUS	Persistent undetermined etiology	44 (55.7)
Other determined	Unchanged primary etiology	63 (94.0)
Other determined	Additional clinically relevant mechanism	4 (6.0)

Percentages were calculated for each initial stroke classification Additional clinically relevant mechanisms include the identification of a clinically relevant stroke mechanism in addition to the initial etiologic classification. Categories are not mutually exclusive for large-artery atherosclerosis, small-vessel disease, and other determined strokes because patients could have additional mechanisms identified while retaining the same primary etiologic classification. ESUS, embolic stroke of undetermined source.

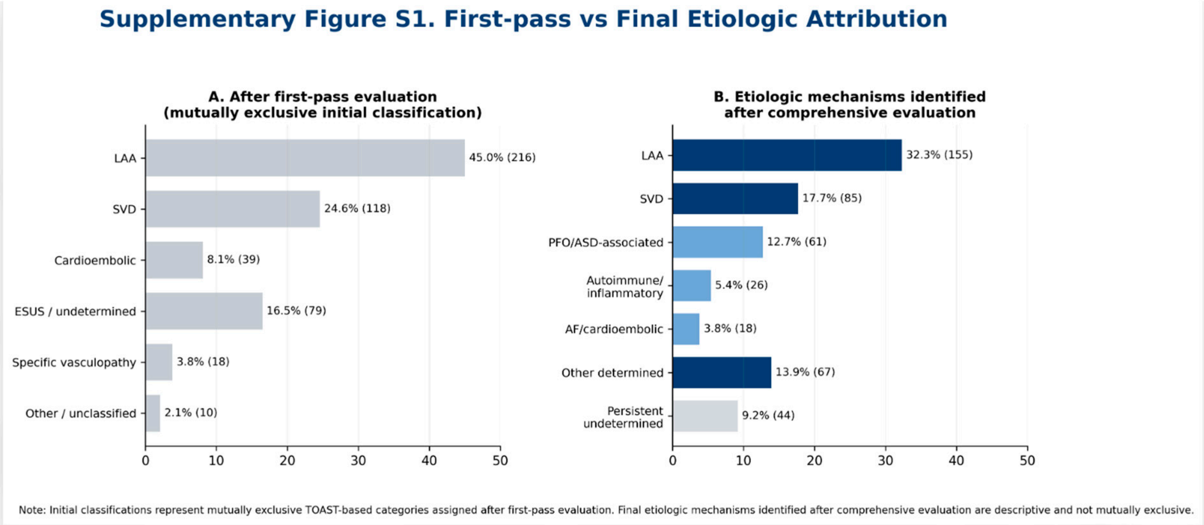

Supplementary Figure S1. First-pass versus final etiologic attribution following comprehensive diagnostic evaluation. Panel A shows the initial mutually exclusive TOAST-based etiologic classification assigned after the first-pass evaluation during index hospitalization. Panel B shows the major etiological mechanisms identified following completion of the comprehensive diagnostic evaluation and longitudinal follow-up. The final categories are descriptive and not mutually exclusive because additional clinically relevant mechanisms could be identified in patients whose primary stroke classification remained unchanged. The proportion of patients with a persistent undetermined stroke etiology decreased from 16.5% (79/480) after the first-pass evaluation to 9.2% (44/480) after completion of the diagnostic pathway. Newly identified mechanisms included PFO/ASD-associated stroke (12.7%), autoimmune/inflammatory stroke (5.4%), and atrial fibrillation–related cardioembolic stroke (3.8%). Abbreviations: TOAST, Trial of Org 10172 in Acute Stroke Treatment; LAA, large artery atherosclerosis; SVD, small vessel disease; ESUS, embolic stroke of undetermined source; PFO, patent foramen ovale; ASD, atrial septal defect; AF, atrial fibrillation.
